# Evaluation of biological and enzymatic quorum quencher coating additives to reduce biocorrosion of steel

**DOI:** 10.1371/journal.pone.0217059

**Published:** 2019-05-16

**Authors:** Siqian Huang, Celine Bergonzi, Michael Schwab, Mikael Elias, Randall E. Hicks

**Affiliations:** 1 Department of Biology, University of Minnesota Duluth, Duluth, Minnesota, United States of America; 2 Department of Biochemistry, Molecular Biology and Biophysics & Biotechnology Institute, University of Minnesota, St. Paul, Minnesota, United States of America; Free University of Bozen/Bolzano, ITALY

## Abstract

Microbial colonization can be detrimental to the integrity of metal surfaces and lead to microbiologically influenced corrosion (MIC). Biocorrosion is a serious problem for aquatic and marine industries in the world. In Minnesota (USA), where this study was conducted, biocorrosion severely affects the maritime transportation industry. The anticorrosion activity of a variety of compounds, including chemical (magnesium peroxide) and biological (surfactin, capsaicin, and gramicidin) molecules were investigated as coating additives. We also evaluated a previously engineered, extremely stable, non-biocidal enzyme known to interfere in bacterial signaling, *Sso*Pox (a quorum quenching lactonase). Experimental steel coupons were submerged in water from the Duluth Superior Harbor (DSH) for 8 weeks in the laboratory. Biocorrosion was evaluated by counting the number and the coverage of corrosion tubercles on coupons and also by ESEM imaging of the coupon surface. Three experimental coating additives significantly reduced the formation of corrosion tubercles: surfactin, magnesium peroxide and the quorum quenching lactonase by 31%, 36% and 50%, respectively. DNA sequence analysis of the V4 region of the bacterial 16S rRNA gene revealed that these decreases in corrosion were associated with significant changes in the composition of bacterial communities on the steel surfaces. These results demonstrate the potential of highly stable quorum quenching lactonases to provide a reliable, cost-effective method to treat steel structures and prevent biocorrosion.

## Introduction

Microorganisms are capable of colonizing surfaces of numerous and diverse materials. This colonization process leads to a firmly adhering and complex microbial community termed biofilm [1]. Biofilms, which can lead to biofouling, are detrimental to their substrates [2, 3] and cause biodeterioration of metal surfaces, known as microbiologically influenced corrosion (MIC) or biocorrosion [4, 5]. Biocorrosion is a severe problem for world maritime industries. Over 20% of all corrosion is associated with biocorrosion, causing an estimated direct cost of 30 to 50 billion US dollars annually [6–8]. Structures in the Duluth-Superior Harbor (DSH; Minnesota, USA), where this study was conducted, are severely affected by biocorrosion. The DSH is located on Lake Superior, the largest reservoir of freshwater in the world. About 20 km of steel sheet piling appear to be affected in the DSH, which may cost more than $200 million to replace [9]. In the past two decades, the severity of steel infrastructure corrosion in the DSH was recognized [9, 10] and the loss of steel in this harbor due to this severe corrosion was estimated be 2 to 12 times greater than in similar freshwater environments [9, 11]. This rate of corrosion suggests there is some accelerating process such as MIC [9, 11, 12]. Among the numerous organisms that colonize the surfaces of metals, sulfate-reducing bacteria (SRB) and iron-oxidizing bacteria (IOB) were previously associated with accelerated biocorrosion rates [12, 13, 14]. Corroding steel pilings in the DSH have a rusty appearance characterized by orange, blister-like, raised tubercles on the surface (S1A Fig) [14]. These tubercles vary in diameter from a few mm to several cm and when removed, large and often deep pits (6 to10 mm) are revealed in the steel, which is sometimes perforated (S1B Fig). This pattern of corrosion is consistent with the appearance of corrosion caused by iron-oxidizing bacteria [13, 14] and sulfate-reducing bacteria [12], and also similar to corrosion of steel structures recently observed at other harbors in Lake Superior.

Corrosion rates in the DSH vary with seasonal temperature changes, which is consistent with biological and chemical processes [10, 11]. Corroded steel surfaces and tubercles in the DSH, like in many other freshwater and seawater environments, are covered by complex microbial biofilms that contain bacteria of the types responsible for corrosion of steel [11, 15–19]. SRB living inside the anoxic zone of the tubercles can either produce hydrogen sulfide, which reacts with iron forming FeS, or directly use iron as electron donor in the metabolism. Also bacteriogenic iron oxides, which are made of a mixture of bacterial cells and amorphous hydrous iron (III) oxide, have reactive porous surfaces and cause enrichment of copper and other heavy metals that could set up electrical currents in corrosion pits under the tubercles. both could accelerate the corrosion process [16, 20].

Numerous strategies have been developed and used to combat biocorrosion [21–24]. In particular, biocidal compounds are widely used [21, 22]. Their relatively low efficacy against biofilm and more importantly their potential environmental hazard makes these compounds undesirable. For example, tributyltin (TBT) was phased out in 2008 due to its detrimental environmental effects, despite its antifouling effectiveness [25]. Therefore, recent efforts have focused on biological or benign molecules to combat biocorrosion to address these ecological and economical concerns [23, 24]. Molecules preventing the adhesion of bacteria or the formation of biofilms have been tested in various coatings and substrates, including metals [21, 24, 26], silicon, organic polymers and glass [24]. Some compounds of biological origin, including antibiotics or bacteria producing antibiotics can impede the attachment of freshwater bacteria and prevent biofouling [27–33]. When mild steel was protected by coatings of biofilm microbes that produce gramicidins, an antibiotic, the steel corrosion rate was reduced 20 times compare to unprotected surfaces [30].

Another approach has recently emerged after the discovery and better understanding of bacterial cell communication. Biofilm production in some bacteria, a key step in the biofouling process, can be regulated by quorum sensing (QS), a mechanism of chemical signaling used by numerous bacteria [34]. QS is the regulation of gene expression in response to fluctuations in cell density. QS bacteria produce and release chemical signal molecules, called autoinducers into their environment [35, 36]. A common class of autoinducers are acyl homoserine lactones (AHLs). Disruption of this communication has been shown to drastically reduce bacterial biofilm formation and virulence for numerous pathogens. A typical approach for disrupting QS consists of using AHL-degrading enzymes called lactonases [37–39]. Through QS disruption, lactonases are capable of inhibiting bacterial virulence and bacterial biofilm formation including in the context of biofouling [16, 40, 41].

In this project, we evaluated the anticorrosion activity of a variety of chemical (magnesium peroxide), biological (surfactin, capsaicin, and gramicidin), and enzymatic (a quorum quenching lactonase) coating additives on steel coupons in DSH water for 8 weeks in the laboratory. We quantified corrosion by counting tubercles and examining ESEM images of samples and determined the microbial community composition on steel coupons coated with a cross-linked silica gel containing the different additives (Table 1).

**Table 1:** List of antifouling biochemical compounds, enzyme and bacteria tested in the experiment.

Antifouling Treatments	Description
Surfactin	A very powerful surfactant commonly used as an antibiotic produced by *Bacillus subtilis*. It prevents biofilm attachment and formation by changing the hydrophilicity of the growth surface [27, 28].
Magnesium peroxide (MgO_2_)	Reduces SRB abundance and sulfate reduction rate under anoxic conditions by decreasing the sulfide concentration in the environment [33].
Capsaicin	An active component of chili peppers. It is cytotoxic to biofilm-forming bacteria [29].
Gramicidin (A, B, and C)	Antibiotic compounds obtained from the soil bacterial species *Bacillus brevis*. They are active against Gram-positive bacteria and select Gram-negative microorganisms [30].
*Sso*Pox W263I lactonase	An improved variant of the hyperthermostable lactonase *Sso*Pox from *Sulfolobus solfataricus*, exhibiting higher catalytic rates. This enzyme degrades acyl-homo N-acylhomoserine lactones (AHLs), the signaling molecules involved in bacterial quorum sensing [31, 32].

## Materials and methods

### Production and purification of the *Sso*Pox W263I lactonase

The *Sso*Pox W263I was produced as previously described [32]. Briefly, *E*. *coli* strain BL21(DE3)-pGro7/GroEL (Takara Bio) was grown in 500 mL of ZYP medium [42] (supplemented with 100 μg/ml ampicillin, 34 μg/ml chloramphenicol) and 0.2% (w/v) arabinose (Sigma-Aldrich) was added to induce the expression of the chaperones GroEL/ES [32]. Lactonase purification was performed as previously explained [32]. Cell lysates with overexpressed lactonase were used as a raw extract. Purified lactonase samples were obtained using a single heating step at 70 °C for 30 min, followed by differential ammonium sulfate precipitation, dialysis and exclusion size chromatography. Pure *Sso*Pox W263I enzyme samples were quantified using a spectrophotometer (Synergy HTX, BioTek, USA) at OD_280nm_ and a protein molar extinction coefficient as calculated by PROT-PARAM (Expasy Tool software) [43]. This purification protocol yielded high purification grade enzyme (>95% purity) [44].

### Steel coupons and silica gel coatings

Steel coupons (5x2x0.95 cm) were cut from hot rolled ASTM-A328 steel, the same material used to construct steel sheet pilings used for most docks and bulkheads in the Duluth-Superior Harbor (DSH). The steel coupons were washed with soapy water (Micro-90, International Products Corp.), lightly brushed for a few seconds with a test tube brush, and then rinsed with Milli-Q water to remove any loose material. Each coupon was designated with a unique number and weighed before being randomly assigned to a specific experimental treatment.

Currently, there are several bio-encapsulation and coating methods for applying anti-corrosion biochemicals or antifouling bacteria onto submerged steel surfaces (e.g., water tanks and ship hulls). Natural polymers are bio-compatible but lack mechanical strength and stability, while synthetic polymers are strong and stable but bio-compatibility is a problem [45]. Here, we used a silica gel coating matrix for the short-term testing of the anti-corrosion biochemicals because of its bio-compatibility and ability to encapsulate biologically active materials [45]. The activity of biochemicals and enzymes can last for as long as several months in silica gel, even after all cells are dead [45]. Silica gel coatings also have the property of not being very durable, which allowed corrosion to occur within the time frame of this study.

The silica gel matrix (a silicon alkoxide cross-linked silica nanoparticle gel), was made by a condensation process (polymerization) of TM40 silica nanoparticles and tetraethoxysilane (Sigma Aldrich Corp. St. Louis, MO, USA) following the procedure of Mutlu et al. [45]. All anti-corrosion biochemicals (surfactin, MgO_2_, capsaicin, gramicidins) except the lactonase enzyme were purchased from Sigma Aldrich. They and the lactonase enzyme were separately added to 15 ml of the silica gel matrix in 50 ml plastic centrifuge tubes to develop the different coatings (Table 2). The exact concentration of each biochemical (20 to 50 μg/ml) was chosen by the concentration that affected biofilm formation or reduced biocorrosion reported by previous research [27–30, 33]. The 100 μg/ml lactonase concentration was determined by preliminary experiments in our labs (S3 Table), which was the lowest concentration that demonstrated the reductions in the number and coverage of corrosion tubercles compared to the control. These coatings were applied by dipping coupons into the appropriate silica gel mixture for 1 min, and then the coating on the coupon surface was air-dried at room temperature for 2 hrs.

**Table 2:** Control and experimental treatments. Control and experimental coupons coated with various anti-corrosion biochemicals or enzyme. Two controls, bare steel (no coating) and coupons with only the silca gel matrix, were used for comparisons to experimental coupons. Each treatment or control had three replicate steel coupons that were in different microcosms. Both control sets of coupons were placed in the same microcosm (#1), and all treatments sets were placed in their own microcosms (#2–6). A total of 6 aquarium microcosms were used in the experiment.

Coupon Set	Treatments	Microcosm #	References
1	Bare steel control without coating	1	
2	Control with crosslinked silica gel coating	1	
3	*Sso*Pox lactonase enzyme (100 μg/ml) in silica gel coating	2	[32]
4	Surfactin (50 μg/ml) in silica gel coating	3	[27]
5	Capsaicin (20 μg/ml) in silica gel coating	4	[29]
6	Gramicidin (50 μg/ml) in silica gel coating	5	[30]
7	MgO_2_ (50 μg/ml) in silica gel coating	6	[33]

### Durability of lactonase-based coatings

We measured the durability of the lactonase containing silica coating exposed to harbor water. Previous reports highlight the extraordinary stability of these enzymes in various conditions, including aqueous environments, and over month periods [46], The silica coating we used is known to partly dissolve after a few weeks [47]. The measured enzyme decay is therefore due to the dissolving of the silica gel in water, leaking of the enzyme from the coating, the degradation of the enzyme, or a combination of all three losses. We used a chromogenic assay [32] to determine the enzyme longevity in the silica gel matrix. While it did not allow us to deconvolute these potential losses, this measure did estimate the durability of the bioactive coating in DSH water. Plastic shaft applicators with 3 mm diameter polyester tips (Thermo Fisher Scientific, USA) were coated with the lactonase treated silica gel coating. The silica gel coating was made using the same method described above. The final *Sso*Pox W263I enzyme concentration was 100 μg/ml. The applicators were dipped into the freshly prepared coating, air dried at room temperature overnight, and then exposed to DSH water in microcosms at 15°C. The lactonase enzyme activity on triplicate enzyme-coated applicators was measured weekly for 6 weeks using a paraoxon enzyme activity assay [32]. Briefly, after each applicator exposed in harbor water was retrieved, it was briefly air dried for few minutes. Then, each applicator was put into 5 mL of 20 mM paraoxon in PTE buffer pH 8 (50 mM Tris, 150 mM NaCl, 0.2 mM CoCl_2_) for 1 hr. Paraoxon hydrolysis was monitored by measuring absorbance at 412 nm with a spectrophotometer (V-1200 Spectrophotometer, VWR International).

### Experimental design and sampling

Five treatments and two controls were investigated to estimate corrosion and changes in bacterial communities on steel coupons (Table 2). Each treatment or control contained three replicate steel coupons that were incubated in separate experimental microcosms constructed from 10-gallon glass aquaria (Aqueon Glass, 50.8 cm × 25.4 cm × 30.5 cm) (S2 Fig). Both control sets of coupons were placed in the same microcosm (#1), and all treatments sets were placed in their own microcosms (#2–6). A total of 6 aquarium microcosms were used in the experiment. Surface water was collected from the Duluth-Superior harbor under the Blatnik Bridge using a bucket one day before the experiment started on June 18, 2015. This water was used to fill each microcosm, which contained an aquarium pump (Aquarium Systems Mini-Jet 404) to constantly circulate the water (~ 2 L hr^-1^) and an acrylic plastic cover with one corner cut out to allow for gas exchange. The coupons in each microcosm were randomly placed in the center of polypropylene scintillation vial racks and immersed in the microcosms for 8 weeks before being recovered for analyses. The microcosms were incubated in a 15°C cold room with continuous florescent lighting during the experiment. The water in each microcosm was exchanged every 2 weeks with fresh surface water collected at the same site in the Duluth-Superior harbor to prevent buildup of decomposition products (i.e. Fe (III) ion) in the water because the microcosms are closed systems [13].

### Corrosion analyses

Biocorrosion was evaluated by measuring the number and coverage of corrosion tubercles. The coupons in each treatment were photographed at the end of the experiment with a digital camera immediately after being removed from the harbor water. Tubercle numbers hand counted and total tubercle area was measured in these digital images using the analyze menu within ImageJ software (NIH, Bethesda, MD, USA). A Hitachi TM-3030 environmental scanning electron microscope (ESEM) was used for the initial surface inspection of the exposed coupons (150X to 300X magnification with 5kV observation condition and standard backscattered electron observation mode) before cleaning. Surface roughness of the steel coupons in each treatment was measured after the exposed coupons were cleaned with the ASTM G1-90 iron and steel chemical cleaning procedure (Stirred vigorously for 2 min in 37% HCl, 50 g/L SnCl_2_ solution at room temperature and brushed the steel surface, then rinse with water and air dried). The ESEM was used again to view details of the cleaned coupon surfaces and the 3D-View software was used to generate average surface roughness measurements (SRa) of the cleaned coupon surfaces using the multi-line scanning method (10 lines per image). The magnification was 200X with 5kV observation condition and standard backscattered electron observation mode. Five randomly selected areas on each coupon were used for calculating surface roughness. Analysis (one-way, non-paired T-tests between each treatment and control) of the tubercle numbers, tubercle coverage, and surface roughness were performed using Microsoft Excel software.

### Microbial analyses

After photographing but before cleaning the steel coupons for ESEM analyses, all material from each control or experimental coupon including biofilm and tubercles was scraped into a sterile 50 ml polypropylene centrifuge tube (Corning, NY USA) using a stainless-steel spatula. DNA in a 0.5 g subsample of this surface material from each coupon was extracted using a PowerSoil DNA kit (MoBio Laboratories, Carlsbad, CA USA). The extracted DNA was used to sequence the V4 region of 16S rRNA gene and describe changes in the composition of bacterial communities. We selected the V4 region of 16S rRNA gene because the Illumina (San Diego, CA USA) MiSeq 2x300 16S rRNA V4 protocol can provide a higher number of good quality overlapped pair-end sequence reads compared to sequencing other 16S rRNA gene variable regions (i.e. V1-V3) [48]. And, the relatively short reads with the 16S rRNA V4 region can still provide good resolution at the order through family taxonomic levels [49]. DNA were quantified with a NanoDrop 2000 Spectrophotometer (Thermo Scientific, Waltham, MA USA) and then used for a qPCR assay and partial 16S rRNA gene sequencing at the University of Minnesota Genomics Center.

Dissimilatory sulfite reductase (*dsr*A) gene qPCR was performed to quantify the abundance of sulfate-reducing bacteria (SRB) in all samples in addition to 16S rRNA gene sequencing. A modified procedure from Kondo et al. [50] was used to estimate the abundance of SRB by quantifying copies of the *dsr*A gene. Quantitative PCR was performed in a 25 μl reaction volume consisting of 12.5 μl Brilliant II SYBR Green Master Mix (Agilent Technologies), 1.0 μl of 10 μM forward DSR-1F (5’- ACS CAC TGG AAG CAC GGC GG -3’) and reverse DSR-R (5’- GTG GMR CCG TGC AKR TTG G -3’) primers, 2.0 μl of 10 mg/ml bovine serum albumin, 3.5 μl of nuclease-free water, and 5.0 μl of DNA template (10 ng total) on a Rotor-Gene 3000 (Corbett Life Science, NSW, Australia) qPCR thermal cycler. A standard curve was developed using *Desulfovibrio vulgaris* subsp. vulgaris genomic DNA (ATCC 29579D-5) amplified with the DSR1F and DSR-R primer set. The standard curve ranged from 0.1 pg to 0.1 ng (400 to 4 x 10^8^ copies of the *dsr*A gene) of this genomic DNA. The qPCR analyses were performed in triplicate on each sample.

### DNA sequence processing and analysis

DNA from microbial communities on the surface of all steel coupons was shipped overnight and sequenced using an Illumina MiSeq at the University of Minnesota Genomics Center, which generated a total of 15,555,272 sequences of the 254 bp portions of the 16S rRNA gene V4 region. Thirty samples were multiplexed in each analysis to theoretically obtain about 500,000 sequences per replicate coupon sample. Illumina sequence data from this study were submitted to the NCBI Sequence Read Archive (SRA) under accession number SAMN09580244.

Sequence data were processed and analyzed using the MOTHUR program [51]. To ensure high quality data for analysis, sequence reads were removed that contained ambiguous bases, homopolymers >7 bp, more than one mismatch in the primer sequence, or an average per base quality score below 25. Sequences that only appeared once in the total set were assumed to be a result of sequencing error and also removed before further analysis. Chimeric sequences were also removed using the UCHIME algorithm within the MOTHUR program [52]. After sequence quality control and chimera removal, the total data set was reduced to 7,590,591 sequences and used for bacterial taxonomy and community analyses. The number of sequences remaining for each coupon after quality control ranged from 123,203 to 492,370. The number of sequences for each replicate coupon was normalized by taking a randomly selected subsample of 123,203 sequences to control for differences in the number of original sequence reads while still capturing as much diversity as possible.

These remaining sequences were clustered into operational taxonomic units (OTUs) at a cutoff value of ≥ 97%. Taxonomy was assigned to OTU consensus sequences by using the Ribosomal Database Project (RDP) taxonomic database. MOTHUR was also used to generate a Bray-Curtis dissimilarity matrix and calculate coverage. Bacterial communities from different samples were compared using ANOSIM, a nonparametric procedure that tests for significant differences between groups, using Bray-Curtis distance matrices in MOTHUR. Bacterial communities on tubercles from different treatments were compared using nonmetric multi-dimensional scaling (NMDS) ordinations in the program PC-ORD (MJM Software Designs, Gleneden Beach, OR USA).

## Results and discussion

### Longevity of the bioactive silica gel coating

One challenge of using enzymes is their lack of stability in different environmental conditions. We used the *Sso*Pox-W263I lactonase enzyme, an engineered variant, in this experiment because of its high catalytic efficiencies against lactones and its extraordinary capacity to withstand harsh chemicals, proteases, and bacterial contents [32, 37, 39]. The enzyme is stable over a wide range of temperatures from -18°C to >70°C and can stay active for > 9 months [46]. Despite its intrinsic high stability, the lactonase *Sso*Pox W263I may denature over time from the action of environmental proteases [46].

The enzymatic activity of the silica gel coating was greatly reduced after 42 days of exposure in DSH water, with a ~50% loss of activity during each week of exposure (S3 Fig). We are unable to deconvolute the relative contributions of any loss of the silca gel coating, leaking of the enzyme from the silica gel coating, or the potential degradation of the enzyme. The weak durability of this coating was a desired property for this study because it allowed for the development of biocorrosion within the time frame of the experiment. Interestingly, significant reductions in corrosion tubercle formation were observed on the coated steel coupon surfaces after 8 weeks despite the rapid decay in lactonase activity in the coating (only 30% remaining after 2 weeks). This finding indicates that most of the action of the lactonase enzyme occurred in the early stage of bacterial surface colonization and corrosion.

### Quorum quenching lactonase-containing coating showed the greatest corrosion reduction

**Fig 1:**
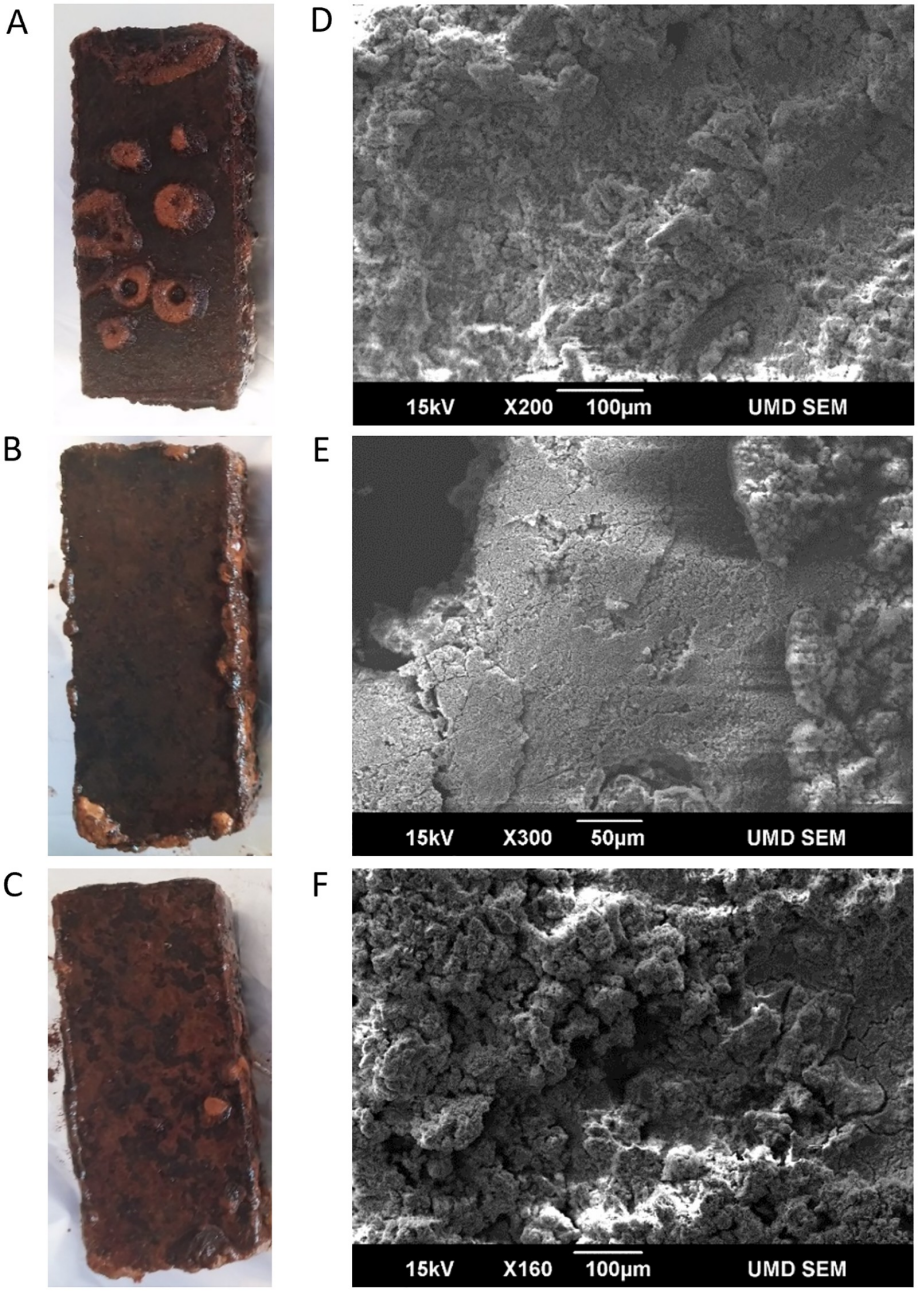
Photographs (A, B, and C) and ESEM images (D, E, and F) of experimental steel coupons after exposure to DSH water for 8 weeks. A, D: Silica gel only control. B, E: Lactonase silica gel treatment. C, F: Surfactin silica gel treatment. Inspection of experimental steel coupons were made at similar magnifications (150x-300x). Occasionally, peeling of the coating (i.e. upper left corner of image E) was observed in each treatment. This is consistent with the nature of the silica gel coating that dissolves after several weeks in aqueous environments [45, 47].

**Fig 2:**
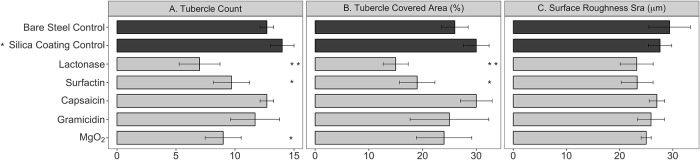
Number and percent coverage of corrosion tubercles, and surface roughness measurements for steel coupons from different experimental treatments. Mean values and standard deviations are shown (n = 3). Means followed by one or two asterisks were different from the silica gel coating control at the significance levels of p<0.05 or p<0.01, respectively. There were no differences in surface roughness (SRa) between any treatments (p’s>0.05).

Corrosion occurred on the steel coupons within 8 weeks of submersion in the DSH water (Fig 1). Corrosion tubercles formed and grew on the steel surfaces, and the silica gel coating alone did not prevent the growth of tubercles (Fig 2). While the use of a weak coating matrix was desired so that corrosion could occur within the time course of the experimental setup, we noted during the initial inspection that the silica gel coating was significantly peeled off (i.e. Fig 1E) by the end of the experiment in all treatments.

There were various amounts of corrosion of the steel coupons in different treatments illustrated by the number and percent coverage of corrosion tubercles (Fig 2). However, the observed corrosion reductions compared to the silica gel coating control were statistically significant for only a few additives, surfactin, MgO, and the lactonase enzyme for the tubercle count, and only surfactin and lactonase for tubercle coverage. The surfactin treatment reduced the number and coverage of tubercles on steel coupon surfaces (31, and 37%, respectively). The lactonase additive gave the strongest corrosion reduction, with the number and coverage of tubercles both being reduced by ~50%. It should be noted that the lactonase concentration used in this study was higher than the concentrations of other tested chemicals because the enzyme is not a biocidal molecule, it only interferes with microbial chemical signaling, and this concentration of lactonase was previously shown to inhibit biofilm formation [37, 40, 53, 54]. We note that due to the use of low concentrations, observed effects of the tested additives are likely to relate to specific effects on bacteria rather than changes in the coating properties. While the efficacy of surfactin on biofouling and biocorrosion has been previously documented [27, 28], the ability of lactonase enzymes to inhibit corrosion has only been suggested [23, 55] but not experimentally verified. Quorum quenching (QQ) approaches, in particular using AHL lactonases, have been studied but focused on the QS systems of gram-negative bacteria and are known to inhibit biofilm formation [56]. The effect of QQ enzymes on more complex bacterial communities is unclear. Yet, reports demonstrate the ability of QQ enzymes, and particularly lactonases, to have a biological effect beyond isolated gram-negative bacterial stains including the inhibition of biofouling [57, 58]. Specifically, the ability of heterologously expressed lactonases to decrease fouling in membrane bioreactors was repeatedly demonstrated [41, 56–58]. In this study, we demonstrated the ability of a purified, extremely stable lactonase to inhibit biocorrosion over a period of 8 weeks in a complex aquatic microbial community.

### All coating additives including the quorum quenching lactonase enzyme changed the microbial community on the steel surface

**Fig 3:**
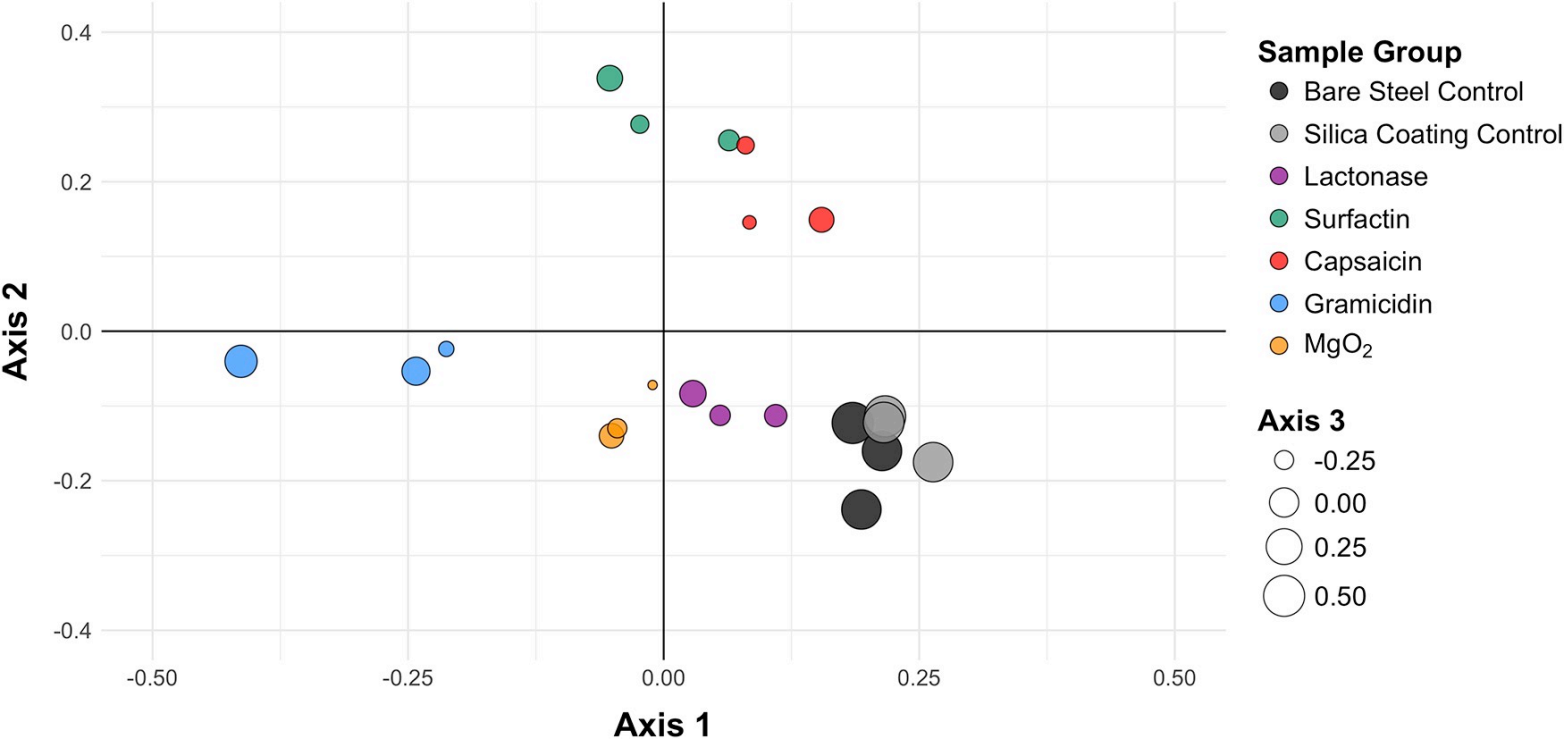
Nonmetric multidimensional scaling plot showing the differences between bacterial communities in different treatments on corroding steel coupons. Each treatment group has data points representing microbial communities from triplicate experimental coupons.

16S rRNA gene sequence analysis (Fig 3) revealed different bacterial communities developed on coupons in all treatments. Each treatment group had data points representing microbial communities from triplicate experimental coupons. The NMDS analysis showed that the different treatments were well separated, with a lowest stress value of 0.16 and an R-squared value of 0.89. The ANOSIM test (S1 Table) indicated that differences between bacterial communities on experimental and control coupons were significant (p<0.05) for all treatments. Conversely, there was no statistical difference between the communities on the two types of control coupons (p = 0.611), suggesting that the silica gel coating had no significant effect on the composition of bacterial community on the steel surface. While it was anticipated that biocidal compounds within the silica gel coating might have an effect on bacterial community composition at the steel surface, it is intriguing that the lactonase enzyme treatment also altered the microbial composition at the surface. Lactonases, and the one used in this study in particular, are not biocides and have no demonstrated effect of bacterial growth [37, 57–59]. Changes in microbial communities induced by lactonase enzymes were also observed in a recent report about a membrane bioreactor [59]. Interestingly, while coating additives that reduced biocorrosion (e.g. surfactin and lactonase) induced changes in the surface microbiome, other tested molecules (e.g. gramicidin, capsaicin) also altered the surface microbial community without significantly affecting the extent of corrosion. Therefore, specific targeting of microbes within the surface microbiome might be necessary for maximal corrosion inhibition.

**Fig 4:**
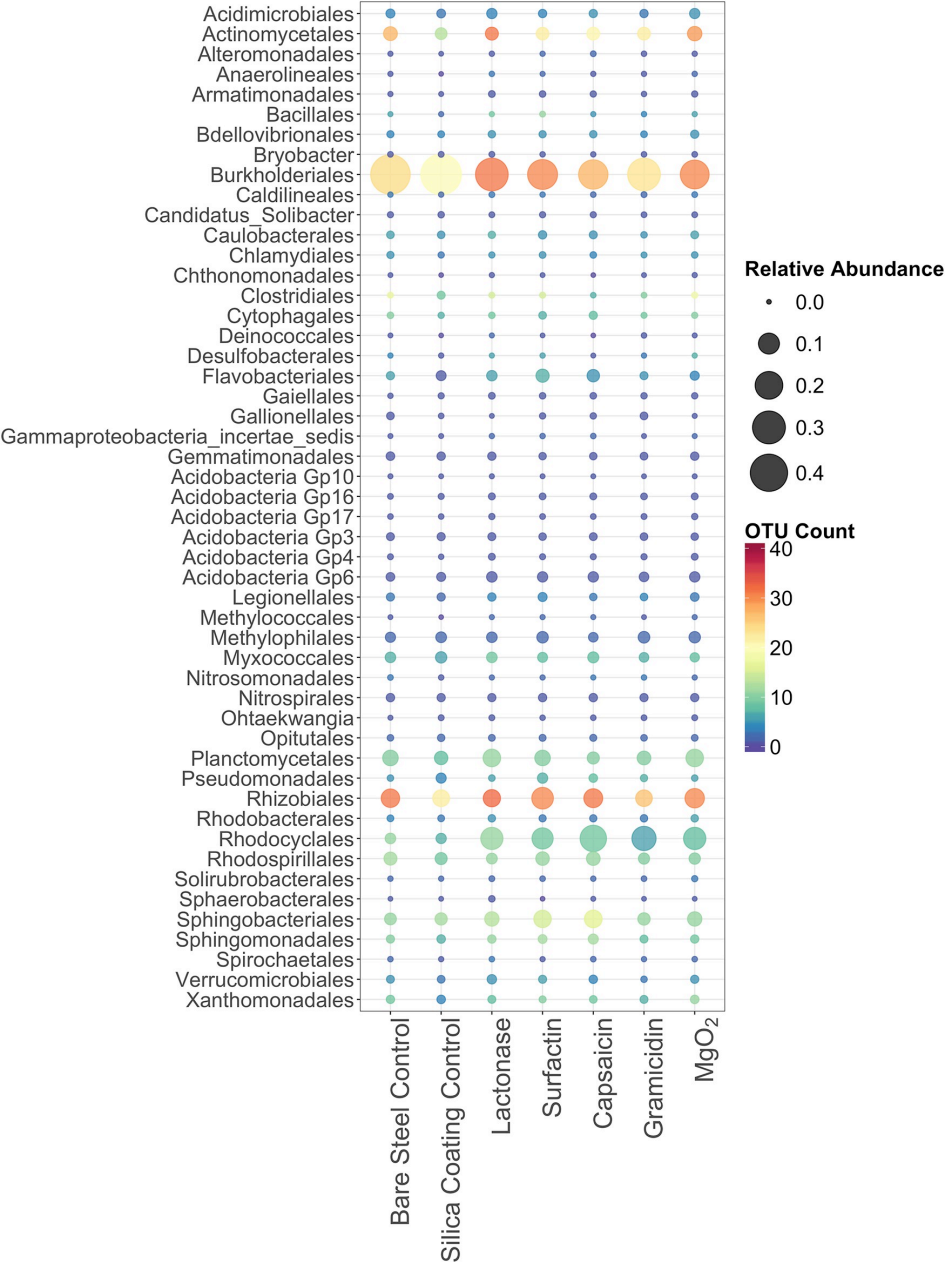
Heatmap comparing the relative abundance of partial 16S rRNA sequences and OTU richness for the top 50 bacterial orders in all samples from each treatment and control. Diversity is indicated by the number of OTUs in each bacterial order.

**Fig 5:**
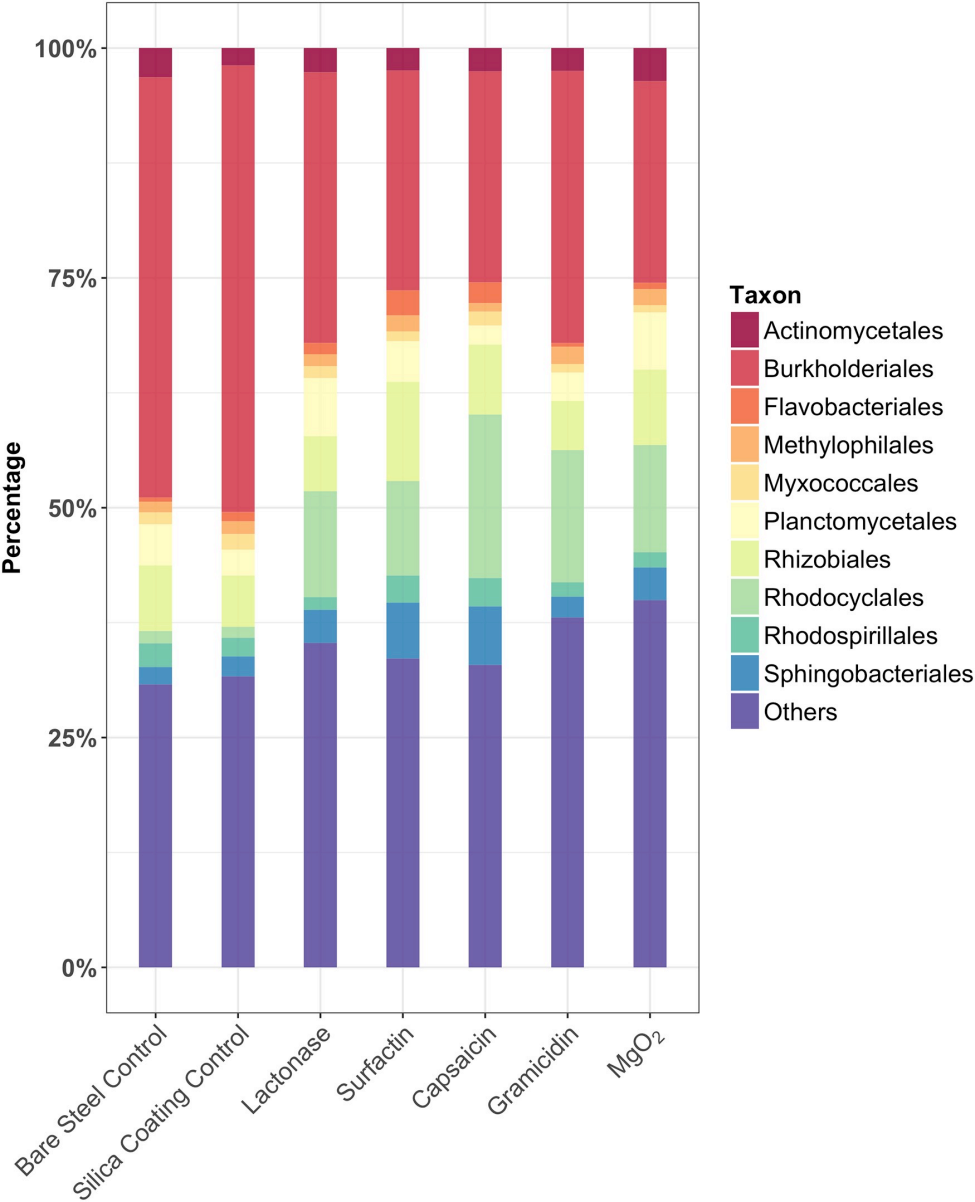
Bar graph showing the community composition of the 10 most abundant bacterial orders by total sequence counts for all treatment and control samples.

A heatmap based on the relative abundances and diversity of sequences from the top 50 bacterial orders (Fig 4), and a bar graph representing relative community composition of top 10 bacterial orders were used to compare bacterial communities in the different treatments (Fig 5). The full heatmap and bar graph comparing the triplicate samples for each treatment is shown in S4 and S5 Figs. Members of the *Burkholderiales* (30%), *Rhodocyclales* (8%) and *Rhizobiales* (8%) were the dominant bacteria found on all coupons. Certain orders of bacteria such as the *Burkholderiales*, *Pseudomonadales* and *Rhodospirillales* were greatly reduced in both the lactonase and surfactin treated samples compared to the silica gel control (S2 Table). Members of the *Burkholderiales* are known to be able to oxidize iron, which has been reported to accelerate corrosion of iron [60–62]. The *Rhodospirillales*, and *Pseudomonadales* are known to contain families of iron oxidizing bacteria, to produce polysaccharides, and accelerate biofilm formation [63]. Sequences from the *Rhodocyclales*, which have previously been identified to contain families of iron-reducers [64], increased in relative abundance in all treatment samples comparing to the controls. These results might indicate that reductions in the number and area of tubercles in the lactonase and surfactin treatments could be caused by differences in bacterial community composition within surface biofilms and corrosion tubercles. Although sequences from SRB orders such as *Desulfobacterales*, *Desulfuromonadales* and *Desulfovibrionales* were found in all samples, their relative abundances were very low (<0.1%) compared to bacterial orders containing iron oxidizers. Within the time course of the experiment no significant corrosion pits, where anaerobic SRB are most likely to be found, developed on the coupon surfaces. Also, the *dsr*A gene was not detected in any microbial community DNA samples by qPCR. This result was consistent with a previous steel corrosion study that demonstrated 16S rRNA sequences from SRB-containing families were rare on coupons exposed in the DSH for less than a year while SRB containing families were more common on steel coupons exposed for more than 6 years, especially in corrosion pits that had formed [15]. This study suggests that the early phase of biocorrosion was not driven by SRB. The lack of SRB presence could explain why the surface roughness differences were not significant despite significant differences in tubercle counts and coverage between the controls and some treatments.

## Conclusions

The potential of various compounds, including a quorum quenching lactonase, to inhibit biocorrosion were evaluated. We found that biocidal molecules such as surfactin and magnesium peroxide were capable of preventing the corrosion process that can be microbiologically influenced. More surprisingly, an extremely stable, engineered lactonase enzyme additive reduced corrosion and exhibited the greatest corrosion inhibition in our study. This finding is intriguing because, in contrast to the biocidal molecules that are dominating the antifouling and anticorrosion coatings market [21, 22], this enzyme has been shown to be non-toxic. In addition to being biodegradable, lactonase proteins show no toxicity [54, 55], and little to no effect on bacterial fitness [46, 52,65], do not need to enter microbial cells, and do not need to bind to a receptor [56, 66]. They act instead by hydrolyzing signaling molecules secreted into the medium to affect bacterial behavior and inhibit biofilms [38, 39, 67], including the biofilms of complex microbial communities [55]. These properties differ from any molecules currently available in coatings, and make lactonases an appealing candidate to the development of potent and environmentally friendly coatings.

Our study also revealed that corrosion inhibition observed for the lactonase and surfactin additives was concomitant to significant changes in the bacterial community composition, which were different and distinct from the changes induced by the other the molecules tested that showed no corrosion inhibition. This outcome indicates that favoring or disfavoring specific microbes at steel surfaces may provide a promising strategy to maximize the inhibition of corrosion while limiting impacts on other microbes and the environment. More studies will be necessary to fully understand the effects of coating additives, including lactonases, on microbial communities involved in biofouling and biocorrosion.

## Supporting information

S1 Fig Examples of corrosion observed in the Duluth-Superior Harbor. A: Orange corrosion tubercles below the water line on a steel piling at the Midwest Energy dock. B: Severe corrosion that has perforated the sheet steel near Hallett Dock 5, which was exposed during low water levels in 2007.
(TIF)

S2 Fig Schematic of an experimental microcosm. The coupons were randomly distributed in the center of plastic scintillation vial holders. Each microcosm was incubated in a 15°C temperature-controlled room with continuous florescent lighting during the experiment. Each microcosm was equipped with an aquarium pump to constantly circulate the water (~ 2 L hr^-1^).
(TIF)

S3 Fig Changes in *Sso*Pox lactonase enzymatic activity in the silica gel coating during exposure in DSH water at 15°C for 6 weeks. The enzyme concentration used in this experiment was 100 μg /ml.
(TIF)

S4 Fig Heatmap comparing the relative abundance of partial 16S rRNA sequences and OTU richness for the top 50 bacterial orders in the replicate samples (n = 3) for each treatment and control. (TIF)

S5 Fig Bar graph showing the community composition of the top 10 most abundant bacterial orders by total sequence counts in each treatment and control triplicate samples. (TIF)

S1 Table Results of ANOSIM test for differences between bacterial communities on experimental and control coupons. (DOCX)

S2 Table List of top 5 percent reduction in relative abundance for the top 20 bacterial orders in lactonase and surfactin treatment samples compared to the silica gel coating control samples. (DOCX)

S3 Table Preliminary experiment results for lactonase dosage response. Reduction in number and percent coverage of corrosion tubercles, and surface roughness measurement on steel coupons with different lactonase concentrations after exposure to Duluth-Superior Harbor water for 6 weeks.
(DOCX)
