# Correction: Evaluation of biological and enzymatic quorum quencher coating additives to reduce biocorrosion of steel

**DOI:** 10.1371/journal.pone.0253354

**Published:** 2021-06-10

**Authors:** Siqian Huang, Celine Bergonzi, Michael Schwab, Mikael Elias, Randall E. Hicks

The Competing Interests statement is incorrect. The correct Competing Interests statement is as follows: ME is the co-founder, Scientific Advisory Board member, and equity holder of Gene&Green TK, a company that holds the license to the patent WO2014167140 A1. These interests have been reviewed and managed by the University of Minnesota in accordance with its Conflict of Interest policies. This does not alter our adherence to PLOS ONE policies on sharing data and materials. The remaining authors declare that the research was conducted in the absence of any commercial or financial relationships that could be construed as a potential conflict of interest.
