# Supplementary figures and images for: Evaluation of biological and enzymatic quorum quencher coating additives to reduce biocorrosion of steel
Source: PLoS One. 2019 May 16;14(5):e0217059. doi: 10.1371/journal.pone.0217059 (PMC6522020; doi:10.1371/journal.pone.0217059)

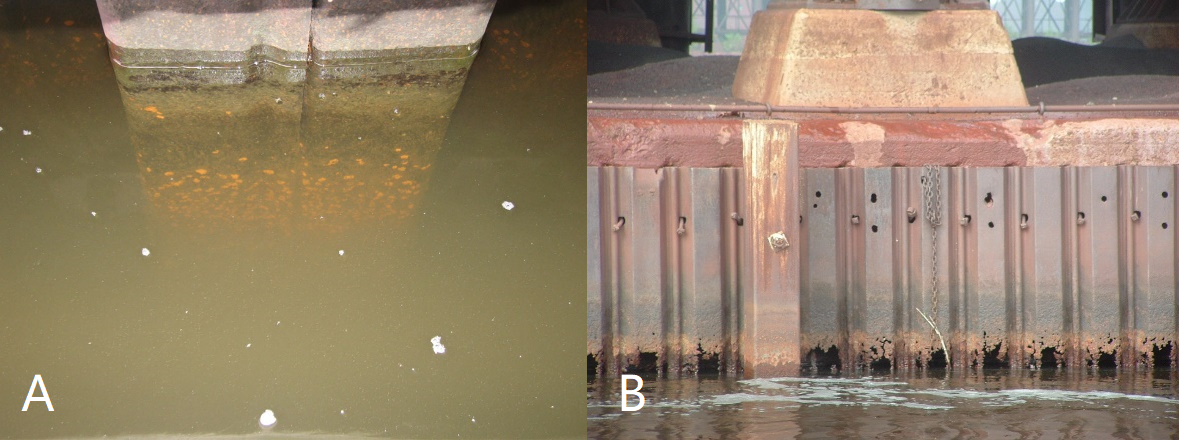

Supplement: S1 Fig — A: Orange corrosion tubercles below the water line on a steel piling at the Midwest Energy dock. B: Severe corrosion that has perforated the sheet steel near Hallett Dock 5, which was exposed during low water levels in 2007. (TIF) [file pone.0217059.s001.tif]

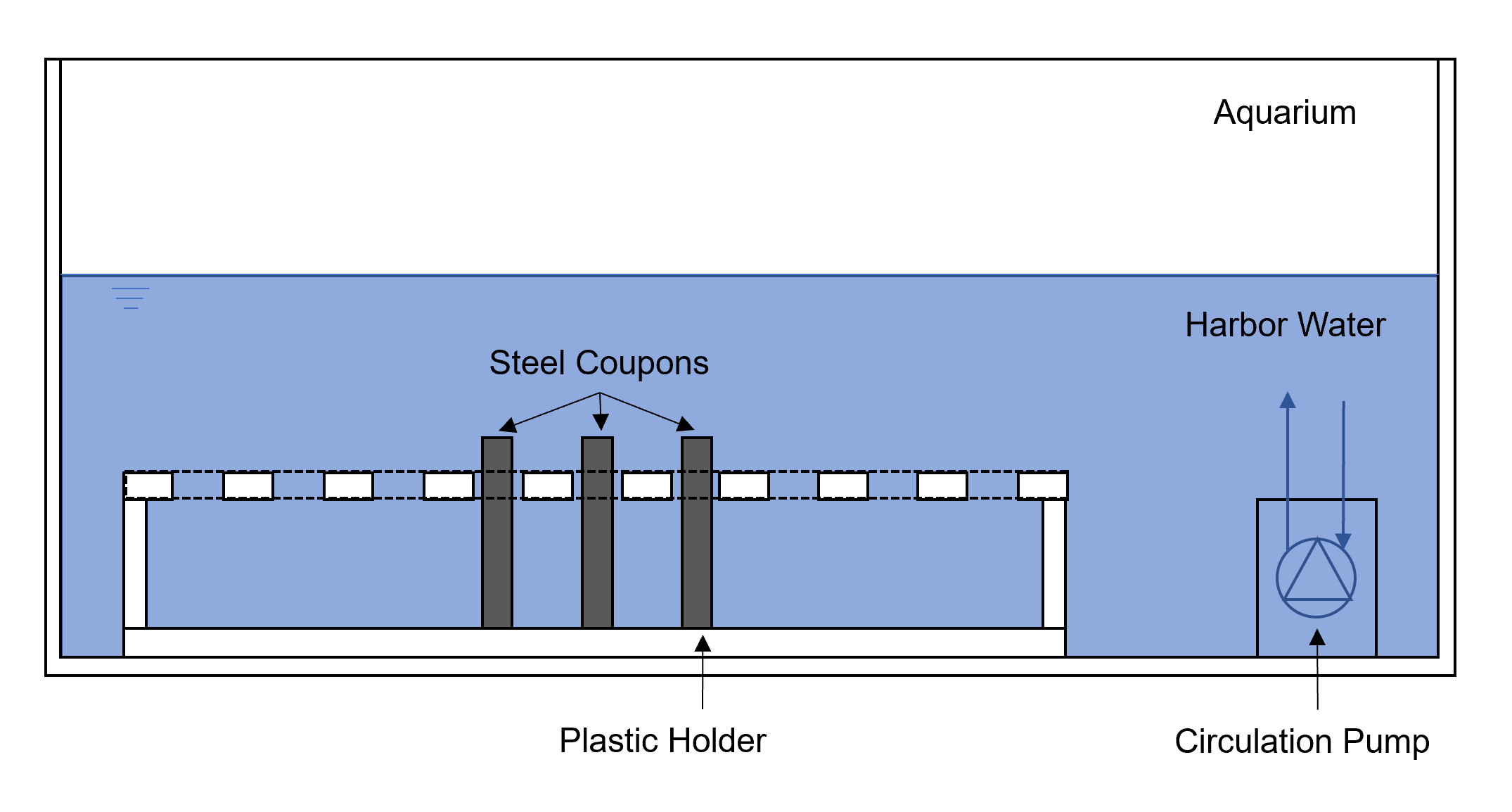

Supplement: S2 Fig — The coupons were randomly distributed in the center of plastic scintillation vial holders. Each microcosm was incubated in a 15°C temperature-controlled room with continuous florescent lighting during the experiment. Each microcosm was equipped with an aquarium pump to constantly circulate the water (~ 2 L hr-1). (TIF) [file pone.0217059.s002.tif]

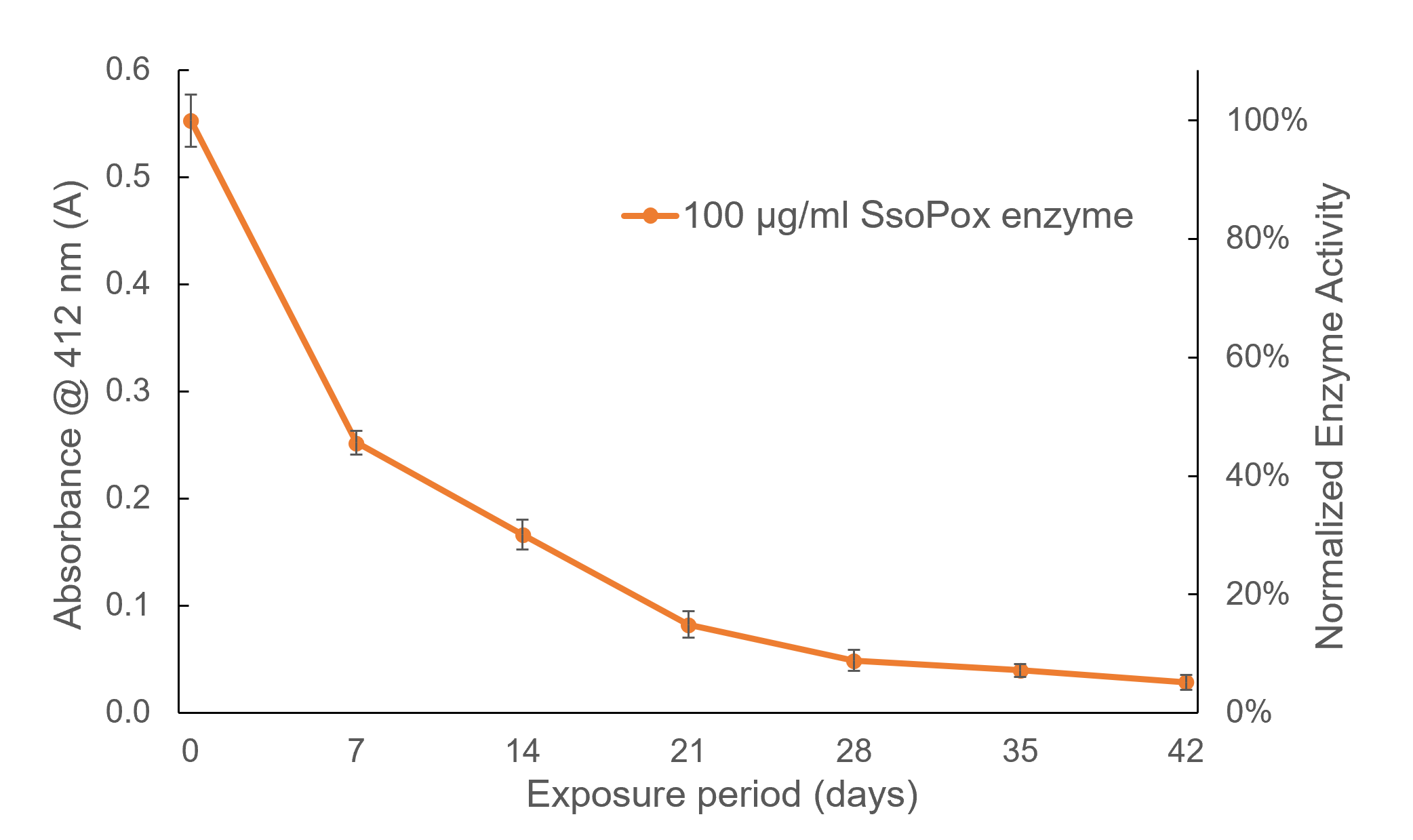

Supplement: S3 Fig — The enzyme concentration used in this experiment was 100 μg /ml. (TIF) [file pone.0217059.s003.tif]

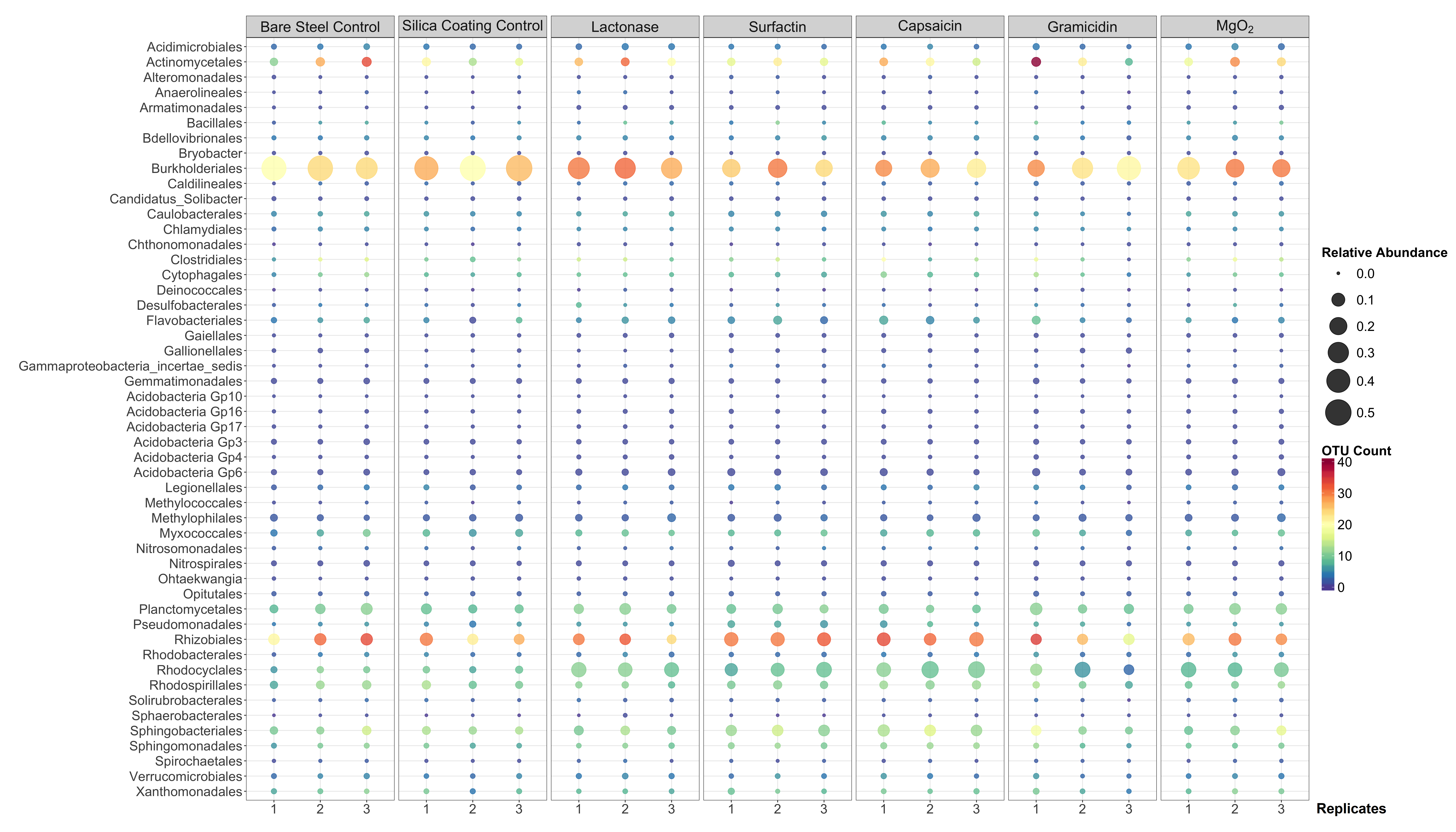

Supplement: S4 Fig — (TIF) [file pone.0217059.s004.tif]

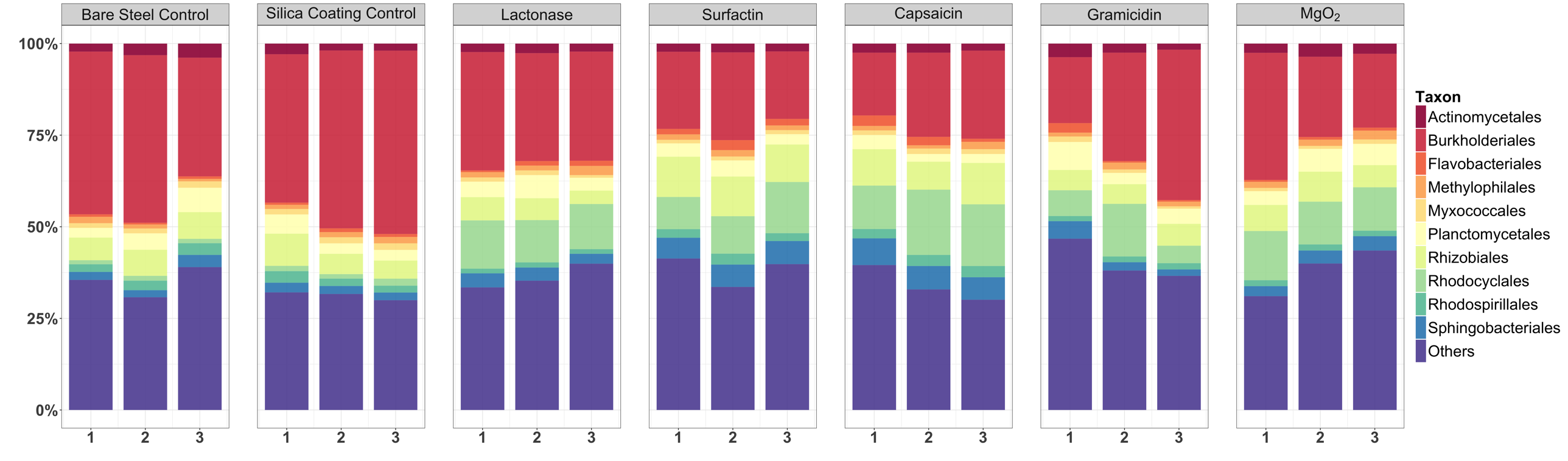

Supplement: S5 Fig — (TIF) [file pone.0217059.s005.tif]
