# Supplementary material for: Evaluation of biological and enzymatic quorum quencher coating additives to reduce biocorrosion of steel
Source: PLoS One. 2019 May 16;14(5):e0217059. doi: 10.1371/journal.pone.0217059 (PMC6522020; doi:10.1371/journal.pone.0217059)
Supplement: S1 Table — (DOCX) [file pone.0217059.s006.docx]

S1 Table. Results of ANOSIM test for differences between bacterial communities on experimental and control coupons.

| **Comparison** | **R-value** | **P-value** |
| --- | --- | --- |
| All groups | 0.72 | <0.001 |
| Capsaicin-Gramicidin | 1.00 | 0.016 |
| Capsaicin-Lactonase | 0.78 | 0.049 |
| Capsaicin-MgO2 | 1.00 | 0.036 |
| Capsaicin-Surfactin | 0.22 | 0.094 |
| Capsaicin-Bare_steel | 1.00 | 0.05 |
| Capsaicin-Coating_control | 0.89 | 0.033 |
| Gramicidin-Lactonase | 0.74 | 0.013 |
| Gramicidin-MgO2 | 0.63 | 0.017 |
| Gramicidin-Surfactin | 0.93 | 0.019 |
| Gramicidin-Bare_steel | 1.00 | 0.009 |
| Gramicidin-Coating_control | 1.00 | 0.002 |
| Lactonase-MgO2 | 0.22 | 0.093 |
| Lactonase-Surfactin | 0.74 | 0.053 |
| Lactonase-Bare_steel | 0.33 | 0.049 |
| Lactonase-Coating_control | 0.44 | 0.045 |
| MgO2-Surfactin | 0.78 | 0.045 |
| MgO2-Bare_steel | 0.74 | 0.036 |
| MgO2-Coating_control | 0.78 | 0.048 |
| Surfactin-Bare_steel | 0.89 | 0.024 |
| Surfactin-Coating_control | 1.00 | 0.009 |
| Bare_steel-Coating_control | -0.04 | 0.611 |
