# Supplementary material for: Evaluation of biological and enzymatic quorum quencher coating additives to reduce biocorrosion of steel
Source: PLoS One. 2019 May 16;14(5):e0217059. doi: 10.1371/journal.pone.0217059 (PMC6522020; doi:10.1371/journal.pone.0217059)
Supplement: S2 Table — (DOCX) [file pone.0217059.s007.docx]

S2 Table. List of top 5 percent reduction in relative abundance for the top 20 bacterial orders in lactonase and surfactin treatment samples compared to the silica gel coating control samples.

| **Top 5 orders** | **Lactonase vs control reduction** | **Taxonomy** | **Surfactin vs control reduction** |
| --- | --- | --- | --- |
| *Pseudomonadales* | -59.7% | *Burkholderiales* | -51.9% |
| *Rhodospirillales* | -46.7% | *Gemmatimonadales* | -44.5% |
| *Burkholderiales* | -32.1% | *Myxococcales* | -36.3% |
| *Myxococcales* | -30.8% | *Acidimicrobiales* | -26.1% |
| *Xanthomonadales* | -28.5% | *Xanthomonadales* | -12.7% |
