# Supplementary material for: Evaluation of biological and enzymatic quorum quencher coating additives to reduce biocorrosion of steel
Source: PLoS One. 2019 May 16;14(5):e0217059. doi: 10.1371/journal.pone.0217059 (PMC6522020; doi:10.1371/journal.pone.0217059)
Supplement: S3 Table — Reduction in number and percent coverage of corrosion tubercles, and surface roughness measurement on steel coupons with different lactonase concentrations after exposure to Duluth-Superior Harbor water for 6 weeks. (DOCX) [file pone.0217059.s008.docx]

S3 Table. Preliminary experiment results for lactonase dosage response. Reduction in number and percent coverage of corrosion tubercles, and surface roughness measurement on steel coupons with different lactonase concentrations after exposure to Duluth-Superior Harbor water for 6 weeks.

| **Treatment** | **Treatment Type** | **Mean Value*** | | | |
| --- | --- | --- | --- | --- | --- |
|  |  | Tubercle numbers | Number Reduction (%) | Tubercle area (%) | Area Reduction (%) |
| Steel with Silica Gel Coating | control | 2.7 | - | 5.9 | - |
| 100 µg/ml Lactonase in Silica Gel Coating | experimental | 1.3 ^a^ | 50 | 3.4 ^a^ | 43 |
| 200 µg/ml Lactonase in Silica Gel Coating | experimental | 1.0 ^a^ | 63 | 3.2 ^a^ | 46 |
| 500 µg/ml Lactonase in Silica Gel Coating | experimental | 1.0 ^a^ | 63 | 3.3 ^a^ | 44 |
| 1000 µg/ml Lactonase in Silica Gel Coating | experimental | 1.3 ^a^ | 50 | 3.4 ^a^ | 42.7 |

* Mean values are shown (n=3). Means followed by a letter superscript were significantly different (p<0.05) from the corresponding control value. Reduction of surface roughness (SRa) was not statistically significant for any treatments compared to the control, and thus is not shown here.
